# Dissecting Hierarchies between Light, Sugar and Auxin Action Underpinning Root and Root Hair Growth

**DOI:** 10.3390/plants10010111

**Published:** 2021-01-07

**Authors:** Judith García-González, Jozef Lacek, Katarzyna Retzer

**Affiliations:** 1 Laboratory of Hormonal Regulations in Plants, Institute of Experimental Botany, Czech Academy of Sciences, 165 02 Prague, Czech Republic; garciago.judith@gmail.com (J.G.-G.); jozeflacek9@gmail.com (J.L.); 2 Department of Experimental Plant Biology, Faculty of Science, Charles University, 128 00 Prague, Czech Republic

**Keywords:** PIN-FORMED2, shootward auxin transport, root growth, root hair, sugar, sucrose, dark grown roots, light grown roots, root hair elongation, total root length, gravitropic index

## Abstract

Plant roots are very plastic and can adjust their tissue organization and cell appearance during abiotic stress responses. Previous studies showed that direct root illumination and sugar supplementation mask root growth phenotypes and traits. Sugar and light signaling where further connected to changes in auxin biosynthesis and distribution along the root. Auxin signaling underpins almost all processes involved in the establishment of root traits, including total root length, gravitropic growth, root hair initiation and elongation. Root hair plasticity allows maximized nutrient uptake and therefore plant productivity, and root hair priming and elongation require proper auxin availability. In the presence of sucrose in the growth medium, root hair emergence is partially rescued, but the full potential of root hair elongation is lost. With our work we describe a combinatory study showing to which extent light and sucrose are antagonistically influencing root length, but additively affecting root hair emergence and elongation. Furthermore, we investigated the impact of the loss of PIN-FORMED2, an auxin efflux carrier mediating shootward auxin transporter, on the establishment of root traits in combination with all growth conditions.

## 1. Introduction

### 1.1. Root Trait Establishment Is Highly Plastic and Depends on Growth Conditions

Plants are divided into above- (shoot) and under-ground (root) organs, and each part has a specific role in capturing the crucial components to ensure plant mass production and health. Both parts of the plant are in constant communication with each other and exchange growth substances, also known as phytohormones, and nutrients (light energy converted to sugar from shoot to root, water and minerals from root to shoot). External signals upon environmental changes are perceived at the cell surface and trigger changes in plant architecture [1,2,3]. Plant roots are very plastic and can adjust their tissue organization and cell appearance during abiotic stress responses [4]. The root consists of a meristematic zone that continually delivers new cells, and its activity arrests when the environmental conditions are not beneficial for the plant. After leaving the division zone, cells pass through the transition and elongation zone towards the differentiation zone, whereby they are maturing and primed according to the growth conditions of the root and the shoot, which are connected through signaling cascades with each other [5,6,7,8]. Root length is defined by the balance between cell proliferation and cell elongation [1,4]. Roots expand and change their architecture, by forming lateral roots and root hairs, to anchor the plant in the soil and enlarge their surface [1,3,4]. Especially root hairs contribute to an efficient uptake of water and nutrients to maximize plant productivity and their outgrowth is highly regulated by environmental conditions [1,2].

### 1.2. Root Growth Is Orchestrated through Interwoven Signaling Cascades

Plant growth’s plasticity, especially the proliferation rate, depends significantly on carbohydrates gained over photosynthesis [8,9]. However, depending on the wavelength, light is also triggering fast-growth processes over asymmetric auxin distribution to change cell elongation behavior [3] and the root is negatively phototropic, which results in growth away from the light source [1,3]. Roots have evolved a finely interwoven network of signaling cascades to adapt to environmental changes, including tight crosstalk between auxin, sugar, and light signaling to balance root growth toward beneficial surroundings but away from harmful influences [1,2,10,11]. Cell elongation processes in the root allow fast growth or to enlarge root surface through root hair outgrowth, both events are highly dependent on sufficient shootward auxin transport along the epidermis [12,13,14,15]. Interference with proper auxin distribution was reported to impact root architecture [16] and to negatively impact gravitropic responses [17]. The root of *Arabidopsis thaliana* is an established model system to study molecular processes underpinning plant growth adaptation upon changing environmental conditions [1,10,18,19,20,21,22]. All root growth aspects are highly dependent on fine-tuned, active polar auxin distribution through the root tip followed by auxin signaling orchestrating cellular responses [3,11,23,24,25,26]. The auxin efflux transporter PIN-FORMED2 (PIN2) orchestrates root growth but is itself regulated on transcriptional through to the post-translational level by external factors such as nutrient availability or light [26,27,28,29,30]. PIN2 abundance and subcellular distribution are dependent on light growth conditions for both the root and the shoot [25,29,30]. Auxin gradients in the root epidermis, which rise from the meristem towards the elongation zone, are crucial to prime trichoblast cells (root hair cells) [31,32,33,34]. Mutants of key players of auxin signaling and transport show severe root hair morphology, spacing, and length phenotypes [15,35,36,37]. 

### 1.3. Direct Root Illumination Triggers Stress Responses and Inhibits Root Growth

*Arabidopsis thaliana* is one of the most studied model plants, especially in terms of cell biology. Few day-old seedlings grown on agar medium in plates became standard test objects and germination on medium allows easy and clean accessibility of the plants for molecular approaches and microscopy. It is known for over a century that plant roots are negatively phototropic, aiming to grow away from a light source [3,10]. However, only over the past decade, biochemical, genetical, and molecular studies reveal the striking impact of direct root illumination on the establishment of the root system architecture [1,21,22,38,39,40]. Direct root illumination triggers stress responses in the root tip, which result in changes of cell fate establishment, including meristem activity, the transition to the elongation zone, and root hair growth [1,21,22]. Root growth differs under direct root illumination among others because of the elevated production of reactive oxygen species, which modulate growth responses on cellular level in the meristem and root hairs [22,38]. In the study of Silva-Navas et al. 2015, the lab introduces a simple and reproducible solution to cover the plates partially with a black box, the D-root system, which allows the cultivation of seedlings in a way that the shoot is exposed to light, but the root is covered [1]. The dark-grown roots (DGR), in comparison to light-grown roots (LGR), show longer roots due to a more active meristem, the number of lateral roots, differential response to hormonal crosstalk, less dramatic phenotypes to additive stress treatment, and altered root hair plasticity [1,21,22]. 

### 1.4. Sucrose Supplementation to the Growth Medium Triggers Auxin Biosynthesis and Results in Altered Root Trait Establishment 

Another widely used cultivation protocol includes the addition of 1% sucrose to the growth medium, often half strength Murashige and Skoog (MS) medium solidified with 1% agar. Studies investigating the role of sugar supplementation to the plant growth medium revealed that it enhances indole-3 acetic acid (IAA) biosynthesis and responses in the root, which is the main naturally-occurring auxin in plants [9,41]. Furthermore, glucose in the medium altered several root traits, which are commonly used to evaluate the impact of auxin-mediated plant growth responses, including total root length, root hair growth (root hair growth), and gravitropic index [41]. Exogenously applied glucose interferes with auxin signaling and transport, which changed total root length, number root hair, and root growth direction [41]. Endogenous sugars, produced upon photosynthesis in the cotyledons, trigger long-distance signaling cascades to modulate root meristem activity in young seedlings [42]. 

### 1.5. Experimental Design to Dissect the Influence of Common Growth Conditions on Root Trait Establishment 

**Figure 1:**
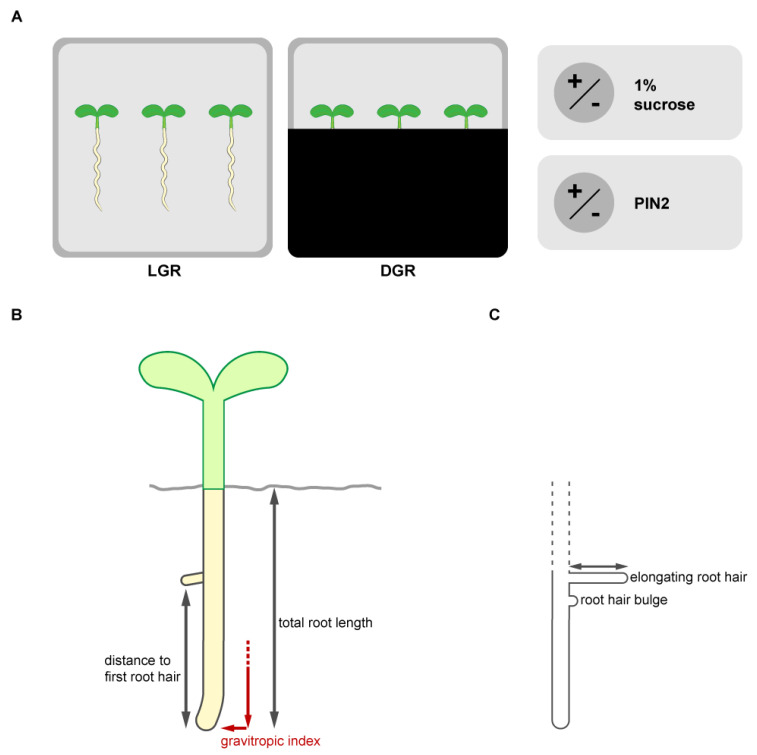
Outline of the experiments. (**A**) To understand the impact of standard lab growth conditions on the outcome of phenotyping experiments we analyzed root traits of seven days old seedlings combining differential root illumination status and sucrose supplementation to the growth medium. To study the influence of direct root illumination the roots were grown in a square petri dish with exposed roots (light grown roots, LGR) and compared to roots grown shaded from light by using the D-root system. The D-root system is a box covering the square plate partially, which allows to grow seedlings with shaded roots, but the shoot stays illuminated, and was introduced by Silva-Navas et al., 2015. To understand the impact of sucrose in the growth medium in combination with altered root illumination status, the seedlings were grown on a commonly used standard medium (half strength MS medium with 1% agar) supplemented with or without 1% sucrose. Sugar supplementation was previously described to enhance the energy level and auxin biosynthesis, which alters root growth. Because shootward auxin transport, mediated by the auxin efflux carrier PIN-FORMED2 (PIN2) in the root tip, is known to be crucial for the establishment of the traits of interest, *Arabidopsis thalilana* lines expressing (*eir1-4 PIN2::PIN2:VEN*) or not expressing (*eir1-4*) PIN2 were compared. Representative pictures of the seedlings grown under all growth conditions are summarized in the Figure S1. (**B**) Root growth traits of the primary root were evaluated (total root length, appearance of the first root hair and gravitropic index). (**C**) Root illumination status, sucrose availability and shootward auxin transport mediated by PIN2 were individually already published to influence root hair emerge and elongation immensely. Therefore, we evaluated the amount of emerged root hairs (bulges and elongated), the percentage of elongated root hairs and root hair length of the elongated root hairs under all growth conditions along the first two mm of the root tip. Representative pictures of the root tips and root hair outgrowth are summarized in the Figure S1.

Here, we describe a timely attempt to dissect the hierarchy of light, sugar and auxin during the growth of roots shaded from direct illumination (Figure 1A). To investigate the interplay of light and sugar we first compared total root length, root hair growth, and gravitropic index (Figure 1B,C) of an established line, expressing wild type levels of the auxin efflux carrier PIN-FORMED2 fused to the yellow fluorescent protein VENUS (PIN2:VEN), driven by the PIN2 promoter [43]. We examined the seedlings seven days after germination (DAG) under four growth conditions, namely DGR with and without 1% sucrose added to half strength MS, and LGR with and without sucrose (Figure 1A). To understand to which extent shootward auxin transport, mediated by PIN2, is involved in the establishment of the chosen root growth traits under the four growth conditions, we compared *eir1-4 PIN2::PIN2:VEN* with the established *pin2* mutant *eir1-4* (SALK_091142) (Figure 1A). Representative pictures of the seedlings grown under all growth conditions are summarized in the Figure S1A and representative pictures of the root hair outgrowth are available in the Figure S1B.

## 2. Results

### 2.1. Direct Root Illumination and Sucrose Supplementation Influence Total Root Length Growth Antagonistically

**Figure 2:**
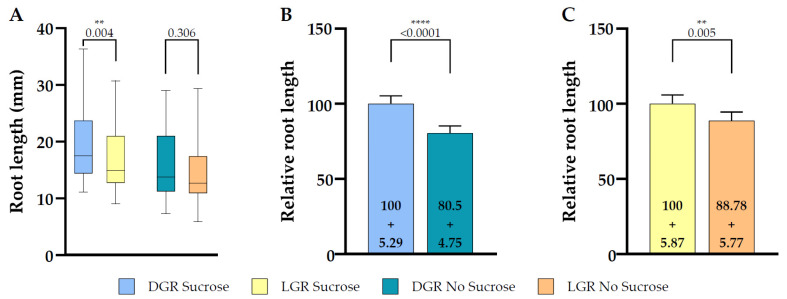
Total root length comparison between dark and light grown 7 DAG *eir1-4 PIN2::PIN2:VENUS* roots in dependence of sucrose supplementation. (**A**) Quantitative analysis of total root length of dark or light grown roots are shown for plants grown on half strength MS medium supplemented with 1% sucrose or without sucrose. (**B**,**C**) Relative root length comparison of seedlings grown under the same root illumination regime, either grown on half strength MS medium supplemented or not supplemented with sucrose, (**B**) for dark grown roots or (**C**) light grown roots (relative length + SE is shown). Differences were assessed by the Mann–Whitney U Test comparing (**A**) dark and light grown roots and (**B**,**C**) plants grown on half strength MS with and without 1% sucrose. p-values are depicted for each comparison (** *p* < 0.01, **** *p* < 0.0001). *n* = 61–79 roots.

The majority of published studies, including those addressing how light, sugar and auxin signaling is modulating root growth, were performed on roots continuously exposed to light, sometimes followed by a shift of the whole seedling to darkness, or on roots of etiolated seedlings. The root is negatively phototropic and direct illumination enhances stress responses that interfere with root growth and responses [21]. Furthermore, the establishment of root traits are also modulated by signals obtained from above ground signals [3]. Root length depends highly on the meristem activity of the root, which is regulated by various signaling cascades integrating environmental signals and availability of resources [1,8]. The D-root system prevents direct illumination of the root and thereby reduces stress responses in the root tip and this results in a higher proliferation rate, the total root length is longer in DGR [1,21]. First we compared if the chosen reporter line *eir1-4 PIN2::PIN2:VEN* responded in the same way as the wild type line *Col-0* used in the original paper introducing the D-root system [1]. We measured total root length of *eir1-4 PIN2::PIN2:VEN* seven DAG grown on half strength MS medium containing 1% sucrose under DGR and LGR conditions, and we observed the expected significant root length difference between the root illumination regimes, with DGR being 19.53 ± SE 0.73/SD 6.49 mm long and LGR 16.71 ± SE 0.69/SD 5.42 mm (Figure 2A).

The composition of growth media immensely influences cell fate and root architecture and because sugar triggers signaling cascades which have an impact on root growth and adaptation processes it is therefore often omitted [41,44]; we compared total root length of *eir1-4 PIN2::PIN2:VEN* seedlings grown seven DAG on medium without sucrose supplementation to understand the impact of sucrose in relation to the used light regimes. Without sucrose the significant difference of total root length was gone between DGR, 15.72 ± SE 0.72/SD 5.74 mm, and LGR, 14.83 ± SE 0.74/SD 5.94 mm (Figure 2A). When we compared the relative total root length upon individual root illumination conditions depending on sucrose supplementation, DGR roots showed a significant difference of total root length (Figure 2B), whereas the difference of LGR is less pronounced (Figure 2C). In conclusion, total root length is inhibited by direct illumination of the root, as it was already described [1]. Furthermore, sucrose has a significant influence by boosting total root length of DGR, indicating that light acts antagonistically to sucrose promoting effect on root length.

### 2.2. Loss of PIN2 Results in Shorter Roots and Counteracts Sucrose Induced Growth Boost of Dark Grown Roots

**Figure 3:**
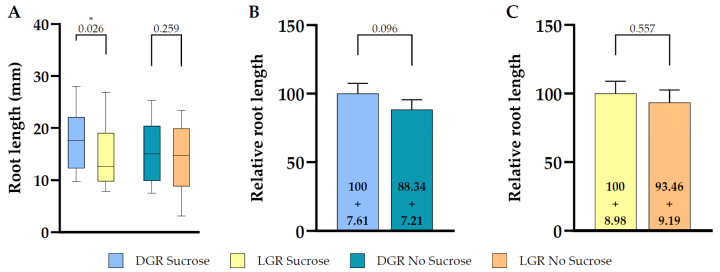
Root length comparison between dark and light grown 7 DAG *eir1-4* roots in dependence of sucrose supplementation. (**A**) Quantitative analysis of root length of dark or light grown roots are shown for plants grown on half strength MS medium supplemented with 1% sucrose or without sucrose. Relative total root length comparison of seedlings grown under the same root illumination regime, either grown on half strength MS medium supplemented or not supplemented with sucrose, (**B**) for dark grown roots (**C**) light grown roots (relative length + SE is shown). Differences were assessed by Mann–Whitney U Test comparing (**A**) dark and light grown roots and (**B**,**C**) plants grown on half strength MS with and without 1% sucrose. *p*-values are depicted for each comparison (* *p* < 0.05). *n* = 34–37 roots.

**Figure 4:**
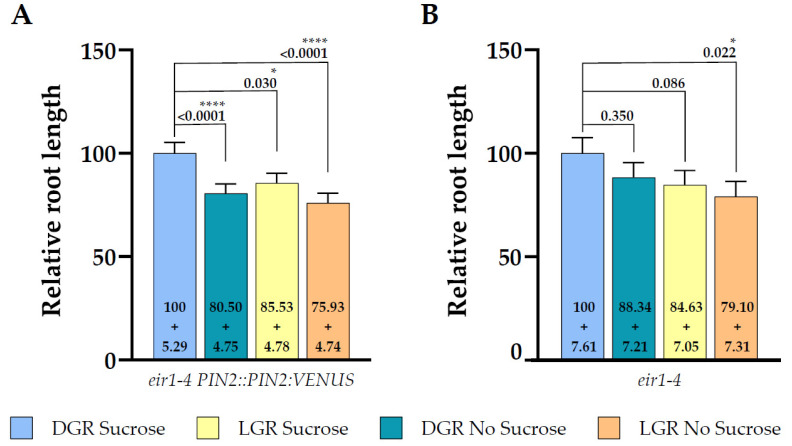
Root length comparison of both lines under all growth conditions relative to dark grown roots germinated on half strength MS supplemented with 1% sucrose. Data shown for (**A**) *eir1-4 PIN2::PIN2:VENUS* and (**B**) *eir1-4* (relative length + SE is shown). Kruskal–Wallis test with Dunn’s post hoc test was used to determine statistical significance. Dark grown roots grown on half strength MS medium supplemented with 1% sucrose were used as reference for the statistical analysis. *p*-values are depicted in each plot (* *p* < 0.05, **** *p* < 0.0001). *eir1-4 PIN2::PIN2:VENUS n* = 61–79 roots; *eir1-4 n* = 34–37 roots.

To understand the impact on total root length of shootward, actively regulated polar auxin transport by PIN2, we compared root length of the PIN2 knockout mutant *eir1-4* under all four growth conditions (Figure 3A). The difference of total root length of DGR seedlings grown on sucrose supplemented medium was less pronounced compared to *eir1-4 PIN2::PIN2:VEN* (Figure 3A; Figure 4), DGR, 17.33 ± SE 0.93/SD 5.52 mm, and LGR, 14.67 ± SE 0.93/SD 5.67 mm. Again, we didn’t observe any significant difference of *eir1-4* seedlings grown on sucrose-free medium between DGR, 15.31 ± SE 0.94/SD 5.48 mm and LGR, 13.71 ± SE 1.03/SD 6.18 mm (Figure 3A). 

When we compared the relative root length ratios within one root illumination regime depending on supplementation, we couldn’t observe any statistically significant change of total root length for *eir1-4* grown on sucrose (Figure 3B) or without (Figure 3C). This is contrasting to the results obtained for *eir1-4 PIN2::PIN2:VEN*, which showed a clear difference of total root length between DGR from sucrose supplemented medium compared to sucrose free medium (Figure 2B). The loss of PIN2 results in overall shorter roots, and dramatically impairs the enhancing effect of sucrose on root length of DGR (Figure 4). Taken together, PIN2 dependent shootward distribution of auxin contributes positive to root length, and the loss of PIN2 resulted in shorter roots under all growth conditions, which correlates with published data showing that *pin2* mutants have a less active meristem and therefore shorter roots [45,46].

### 2.3. Differential Root Illumination Influences the Number of Emerging Root Hairs, and Sucrose Enhances Root Hair Length of eir1-4 PIN2::PIN2:VEN

**Figure 5:**
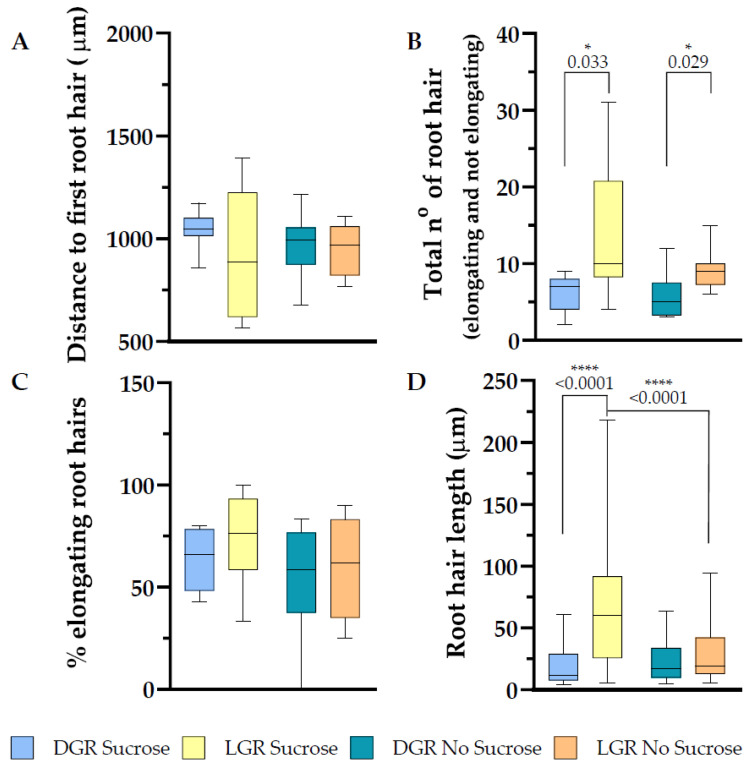
Root hair trait evaluation along the first 2 mm of the root tip of 7 DAG *eir1-4 PIN2::PIN2:VENUS* plants. Depicted are (**A**) the distance from the root tip to the first emerging root hair, (**B**) total amount of root hairs (bulges and elongated), (**C**) in percentage how many root hairs elongated and (**D**) root hair length. (**A**–**D**) Root hair traits were evaluated from dark and light grown roots, grown either on half strength MS medium supplemented with 1% sucrose or without sucrose. Roots measured: *n* = 7–12. Root hairs measured: 25–122. Student’s *t*-test (total number of root hairs and distance to first root hair) or Mann–Whitney *U* test (% of elongating root hairs and root hair length) was used to perform statistical analysis for the following comparisons: LGR sucrose vs. LGR no sucrose; DGR sucrose vs. DGR no sucrose; LGR sucrose vs. DGR sucrose; LGR no sucrose vs. DGR no sucrose. *p*-values for statistically significant comparisons are depicted in each plot (* *p* < 0.05, **** *p* < 0.0001).

Root hair establishment and polar outgrowth is very plastic and sensitively enhanced or inhibited by hormonal and secondary messenger signaling, which are responding to exogenous signals balanced against available resources of the plant [2,32,47]. Direct root illumination negatively correlates with the plasticity of root hairs under phosphate deficient growth conditions [22]. When grown on sucrose supplemented half strength MS medium, LGR possess longer root hairs close to the meristem, which was suggested to result from elevated stress responses in the root tip [1]. We compared the impact of sucrose in combination of the root illumination regime on root hair outgrowth and measured the distance between the bottom of the root columella and the first visible root hair bulge, which is defining the end of the elongation zone of the root, and the beginning of the differentiation zone. *Eir1-4 PIN2::PIN2:VEN* showed no statistically significant change in the length of the meristem and elongation zone combined depending on the growth conditions (Figure 5A), LGR with sucrose 929.6 ± SE 91.87/SD 304.7 μm; LGR without sucrose 950.4 ± SE 43.62/SD 123.4 μm; DGR with sucrose 1043 ± SE 49.13/SD 96.66 μm and DGR without sucrose 971.9 ± SE 56.25/SD 159.25 μm. Further detailed determination of meristem and elongation zone length, cell number, and cell volume will allow to understand if there is a change of root zonation establishment depending of light and sucrose perception. We further focused on the evaluation of root hair traits, and measured the number of all root hairs emerging, as bulges and elongated, along the first 2 mm of the root tip. We measured for DGR, independent on sucrose supplementation, the lowest number of root hairs (6 ± SE 0.93/SD 2.45 for DGR with sucrose, and 5.75 ± SE 1.07/SD 3.01 for DGR without sucrose; Figure 5B), whereas direct root illumination triggered the appearance of root hairs clearly, with no significant enhancement upon sucrose supplementation (14.50 ± SE 2.44/SD 8.46 for LGR with sucrose, and 9.25 ± SE 0.98/SD 2.77 for LGR without sucrose; Figure 5B). 

We evaluated root hair elongation of *eir1-4 PIN2::PIN2:VEN* under all four growth conditions and couldn’t detect a significant difference of the percentage of elongating root hairs, LGR with sucrose 75.20 ± SE 5.99/SD 20.73 %; LGR without sucrose 59.54 ± SE 11.85/SD 24 %; DGR with sucrose 63.8 ± SE 7.53/SD 15.85 % and DGR without sucrose 53.75 ± SE 9.66/SD 27.34 % (Figure 5C). In the study of Silva-Navas et al., 2015 they described that in Col-0 the root hair closer to the meristem were longer under direct root illumination, when using sucrose supplemented medium, we observed the same pattern for *eir1-4 PIN2::PIN2:VEN*, LGR with sucrose 66.29 ± SE 4.16/SD 49.98 μm, and DGR with sucrose 20.47 ± SE 3.31/SD 16.54 μm (Figure 5D). Without sucrose supplementation, the root hair length for LGR 28.37 ± SE 3.26/SD 21.88 μm, was not significantly different to DGR, 23.16 ± SE 3.22/SD 16.43 μm (Figure 5D). Taken together, without any further additive stress treatment, direct root illumination results in elevated root hair emergence, independent on sucrose supplementation, whereas sucrose supplementation triggers an increase in root hair length of LGR, but not upon the other three growth conditions.

### 2.4. Root Hair Outgrowth of eir1-4 Is Strongly Inhibited on Sucrose Free Medium

**Figure 6:**
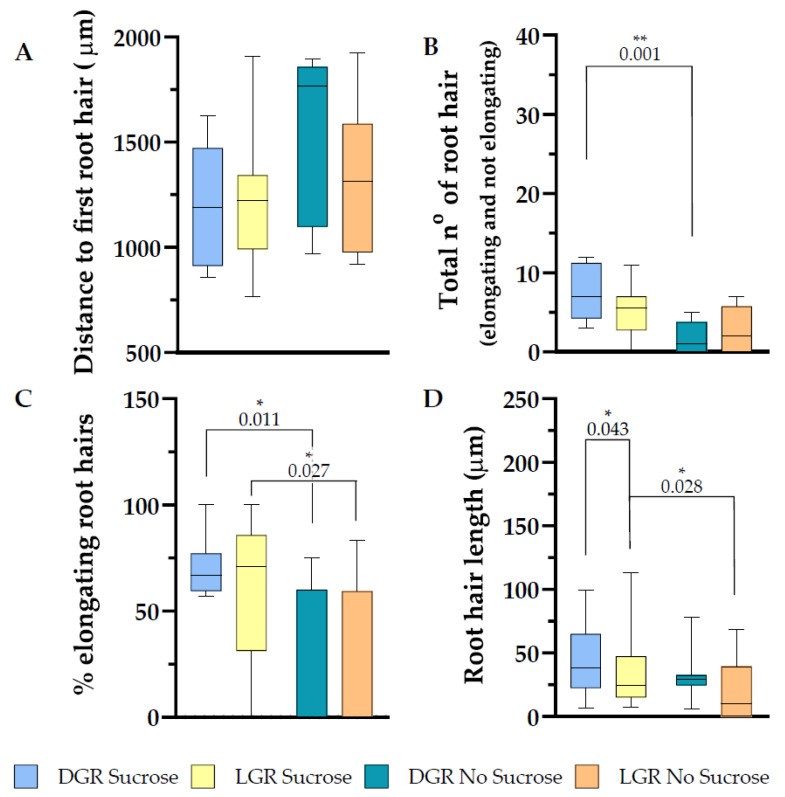
Root hair trait evaluation along the first 2 mm of the root tip of *eir1-4* plants. Depicted are (**A**) the distance from the root tip to the first emerging root hair, (**B**) total amount of root hairs (bulges and elongated), (**C**) in percentage how many root hairs elongated and (**D**) root hair length. (**A**–**D**) Root hair traits were evaluated from dark and light grown roots, grown either on half strength MS supplemented with 1% sucrose or without sucrose. Roots measured: *n* = 8–14. Root hairs measured: 7–45. Student’s *t*-test (total number of root hairs and distance to first root hair) or Mann–Whitney *U* test (% of elongating root hairs and root hair length) was used to perform statistical analysis for the following comparisons: LGR sucrose vs. LGR no sucrose; DGR sucrose vs. DGR no sucrose; LGR sucrose vs. DGR sucrose; LGR no sucrose vs. DGR no sucrose. *p*-values for statistically significant comparisons are depicted in each plot. (* *p* < 0.05, ** *p* < 0.01)

Root hair initiation and elongation depend on proper auxin distribution and signaling along the root and in the individual root hair [15,48,49]. To evaluate the impact of PIN2 action in relation to root illumination and sucrose supplementation, we measured the differences in root hair appearance, number, and percentage of elongating root hairs of root tips 2 mm shootward. Consistent with published data [15,32], root hair appearance and elongation are altered in *eir1-4* compared to *eir1-4 PIN2::PIN2:VEN* control line, which expresses PIN2 at wild type levels. Root hairs appeared further away from the root tip in the mutant compared to *eir1-4 PIN2::PIN2:VEN*, LGR with sucrose 1216 ± SE 74.08/SD 277.2 mm, LGR without sucrose 1321 ± SE 126.3/SD 357.3 mm, DGR with sucrose 1196 ± SE 101.3/SD 286.5 mm, and DGR without sucrose 1553 ± SE 138.4/SD 391.5 mm (Figure 6A). The amount of emerging root hairs dropped along the first 2 mm of root tip in comparison to *eir1-4 PIN2::PIN2:VEN*, which was further more evident when sucrose was omitted in the growth medium, LGR with sucrose 5.14 ± SE 0.79/SD 2.96, LGR without sucrose 2.75 ± SE 1.03/SD 2.92, DGR with sucrose 7.38 ± SE 1.21/SD 3.42, and DGR without sucrose 1.75 ± SE 0.70/SD 1.98 (Figure 6B). *Eir 1–4* responded differently if compared to *eir1-4 PIN2::PIN2:VEN* that showed upon direct illumination a higher emergence of root hairs compared to the DGR. *Eir1-4* didn’t show a difference regarding root hair amount upon different light regimes, supporting published studies stating that root hair outgrowth in general, and further under light stress, is auxin dependent [1,15]. 

Root hair elongation of *eir1-4* changed compared to *eir1-4 PIN2::PIN2:VEN*, regarding the significant difference of the elongation rate depending on sucrose supplementation to the growth medium. *Eir1-4* showed a statistically relevant drop of the percentage of elongating root hairs when sucrose was omitted in the medium, LGR with sucrose 60.98 ± SE 9.06/SD 33.90 %; LGR without sucrose 25.06 ± SE 12.53/SD 35.43%; DGR with sucrose 70.88 ± SE 4.89/SD 13.85 % and DGR without sucrose 22.71 ± SE 11.61/SD 32.83% (Figure 6C). Further studies are imminent to dissect at which developmental stage of the root and to which extent sucrose is triggering root hair growth. From former studies, it can be deduced that sucrose is enhancing auxin biosynthesis in the root tip [9,41], and 1% sucrose elevates levels of IAA by three-fold [9], whereby auxin is required to prime root hair cell fate long before they reach the differentiation zone [15]. Although sucrose is enhancing root hair emergence, root hair length was not promoted further at LGR, contrary to data obtained for *eir1-4 PIN2::PIN2:VEN*. Root hair length for the other three conditions resembled for *eir1-4* values of *eir1-4 PIN2::PIN2:VEN*. The root hair length was for LGR with sucrose 35.28 ± SE 4.37/SD 29.30 μm, LGR without sucrose 22.05 ± SE 6.11/SD 22.84 μm, DGR with sucrose 44.04 ± SE 4.07/SD 24.75 μm, and DGR without sucrose 32.29 ± SE 8.30/SD 1.97 μm (Figure 5D). Taken together, PIN2 modulated shootward auxin transport is crucial to initiate and promote root hair growth, which is consistent with published data. *Eir1-4* root hair phenotypes can be partially rescued by sucrose supplementation, as root hair emerge of DGR was significantly enhanced, and sucrose is slightly shifting the ration from root hair bulges towards elongated root hairs, but the elongation potential as seen for *eir1-4 PIN2::PIN2:VEN* is lost. Root hairs of LGR grown on sucrose supplemented medium elongate more than three times longer in average compared to DGR grown on sucrose supplemented medium (Table S3). 

### 2.5. Sucrose Supplementation Results in More Randomized Vertical Growth of eir1-4 PIN2::PIN2:VEN Independent on Root Illumination, but only in DGR for eir1-4

**Figure 7:**
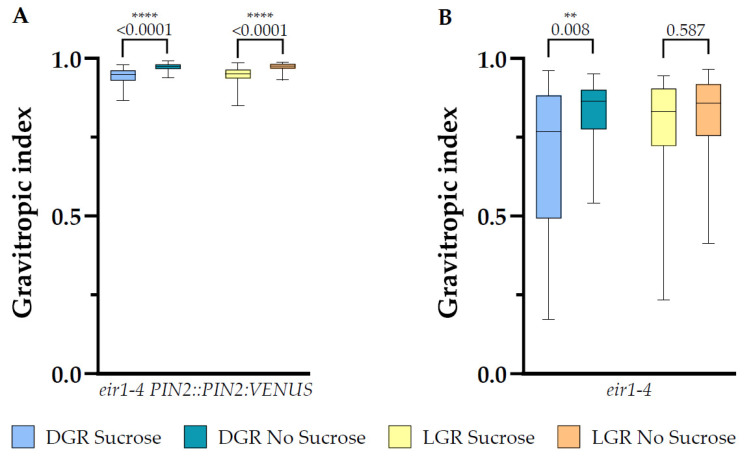
Root gravitropic index analysis of 7 DAG plants. Shown are gravitropic index data plots for (**A**) *eir1-4 PIN2::PIN2:VENUS* and (**B**) *eir1-4* plants grown on half strength MS medium either supplemented with 1% sucrose or without sucrose, of dark, respectively light grown roots. Differences were assessed by. Mann–Whitney *U* test was used to perform statistical analysis comparing plants grown on half strength MS medium with and without 1% sucrose supplementation under specific root illumination conditions (dark and light). *p*-values are depicted for each comparison (** *p* < 0.01, **** *p* < 0.0001). *eir1-4 PIN2::PIN2:VENUS n* = 61–79 roots; *eir1-4 n* = 34–37 roots.

Previously, glucose was reported to lead to randomized root growth, with a stronger deviation from vertical compared to roots grown on medium without sucrose supplementation [41]. Furthermore, upon glucose addition to the growth medium PIN2:GFP was stabilized on lateral PMs and enhanced shootward auxin transport was measured, probably interfering with the fine-tuning of growth along the gravity vector [41]. Increased root tip growth deviation from vertical can is visible in decreased values of the vertical growth index, in our study named gravitropic index [50]. Our analysis of the gravitropic index of *eir1-4 PIN2::PIN2:VEN* roots showed that sucrose is like glucose leading to a small, but highly significant randomization of root growth, which is not dependent on the root illumination regime, LGR with sucrose 0.95 ± SE 0.003/SD 0.03; LGR without sucrose 0.97 ± SE 0.002/SD 0.01; DGR with sucrose 0.94 ± SE 0.003/SD 0.023; and DGR without sucrose 0.97 ± SE 0.001/SD 0.01 (Figure 7A). In the case of the agravitropic *eir1-4*, we could only measure a small statistically significant difference of the gravitropic index for DGR, with sucrose 0.68 ± SE 0.038/SD 0.23; without sucrose 0.83 ± SE 0.018/SD 0.11, whereas there was no difference for LGR, with sucrose 0.79 ± SE 0.027/0.17; without sucrose 0.82 ± SE 0.023/SD 0.14 (Figure 7B). 

## 3. Discussion

Previously published studies linked the root illumination status and sucrose supplementation of the growth medium to altered root trait establishment, including differences in total root length and root hair outgrowth [1,25,41]. The establishment of those root traits depends strongly on fine-tuned polar auxin distribution, mediated by the auxin efflux carrier PIN2, through the root tip and availability of resources delivering energy to maintain proper root growth, in correlation to exogenous stimuli, like changing illumination or nutrition levels [3,11,23,25,26]. With this study, we present a first attempt to dissect the interplay between shootward transported auxin by PIN2, illumination status of the root and sucrose supplementation to the growth medium to mediate root growth and root hair outgrowth. A summary of all data is available in the Tables S1 and S2. Additionally, we calculated the changes of the measured root traits in percentage compared to DGR *eir1-4 PIN2::PIN2:VEN* grown on medium supplemented with sucrose and summarized them in the Table S3. Overall, our observations showed that sugar supplementation and continuous illumination of the root have an antagonistic effect on root length but an additive effect on root hair outgrowth. DGR showed more obvious difference of total root length depending on sucrose availability compared to LGR. The *pin2* mutant showed a similar sensitivity towards root illumination regime, but the total root length difference between DGR and LGR grown on sucrose was less pronounced compared to the control line, and total root length of the mutant was shorter upon all four growth conditions. 

Root hair abundance and length are essential root traits to maximize nutrient uptake and therefore plant productivity, and depend on the transduction of environmental signals through the whole plant body towards the root tip [32,51]. Tubular root hair outgrowth from the epidermis is an example of the planar and polar elongation of a cell and is regulated by various extra- and intracellular signaling events, highly depending on fine-tuned establishment of auxin gradients along the root tip [15]. The distribution of auxin is orchestrated in response to environmental stimuli in an active, directed (polar), cell-to-cell mediated way to define the spacing, abundance, and length of root hairs [32]. Direct, continuous illumination of the root induce stress responses [21] and auxin, light, and sugar signaling interfere with each other in a complex network to modulate meristem activity and root hair development [2]. Therefore, so far, most studies connecting light, sugar and auxin signaling underpinning root hair growth were done by evaluating constantly illuminated roots. This study dissects for the first time, the impact of shading the root from direct illumination in relation to sucrose levels and PIN2 loss during root hair outgrowth. Our results show that direct root illumination of *eir 1-4 PIN2::PIN2:VEN* results in a higher number of root hairs for LGR compared to DGR, independent on the availability of sucrose, but only root hairs of LGR grown on sucrose supplemented medium showed an extended ability for elongation. This ability was lost in *eir1-4*, accompanied by a drastic reduction of root hair emergence, especially if sucrose was omitted in the growth medium, independent on the root illumination status. Taken together, upon PIN2 action direct root illumination results in elevated root hair emergence, independent on sucrose supplementation, whereas sucrose supplementation triggers root hair length of LGR, but it doesn’t for the other three growth conditions. In contrast, when PIN2 activity is lost, sucrose is partially elevating root hair emergence, but not root hair elongation. This indicates that sucrose supports trichoblast priming and partially root hair elongation, but shootward auxin delivery to the individual trichoblast is necessary to enhance its tip elongation. These results correlate with the published data that show that sugar is enhancing auxin biosynthesis in the root [9], but it also shows that for efficient root hair elongation on-point delivery of auxin to the individual root hair is crucial [32,51]. When shootward auxin transport is reduced root hairs fail to elongate, which was shown for the knockout *pin2* mutant [32,51]. Taken together, our data imply that shootward, PIN2 mediated auxin transport is crucial to implement light and sucrose mediated responses to orchestrate root hair elongation plasticity, whereas trichoblast priming is triggered also without PIN2 activity upon light stress in the presence of sucrose. The characterization of the deviation of root growth away from vertical of *eir1-4 PIN2::PIN2:VEN*, showed that sucrose is leading to a small, but highly significant randomization of the root growth direction, which is not dependent on the illumination regime of the root. This result correlates with the published study of Mishra et al., 2009, where they showed that glucose supplementation leads to randomized root growth in comparison to seedlings grown on sugar free medium, and further that glucose stabilized PIN2:GFP at the lateral PMs of some cells and enhanced shootward auxin transport towards the elongation zone, thereby linking sucrose signaling to auxin responses [41]. In contrast to *eir1-4 PIN2::PIN2:VEN*, *eir1-4*, which represents an agravitropic mutant [52], showed only a minor change of the gravitropic index of DGR depending on the availability of sucrose, but we couldn’t measure a significant difference for LGR. Further evaluation of changes on cellular and subcellular level are required to dissect the molecular network connecting auxin, sugar, and light signaling cascades, which are involved in root and root hair growth regulation, root zone establishment, and switch between cell proliferation and elongation.

## 4. Materials and Methods 

### 4.1. Plant Material and Growth Conditions

Seed stocks *eir1-4 PIN2::PIN2:VENUS* and *eir1-4* were obtained from Christian Luschnig, University of Natural Resources and Life Sciences, Vienna, Austria. Seeds were surface sterilized using 50% (*v*/*v*) bleach and 0.1% Tween20 (Sigma-Aldrich, Darmstadt, Germany) for 5 min and then rinsed three times with sterile water. The seeds were plated on 0.5× Murashige and Skoog (Sigma) medium, solidified with 1% agar (Sigma) and adjusted to pH 6.0 by KOH. The medium was supplemented by 1% sucrose (Merck-Millipore, Darmstadt, Germany), or was left sugar free to test the impact of sugar on root growth. Half of the plates were covered by the D-root system [1] to investigate the impact of direct root illumination on root growth. The seeds were plated and stratified at 4 °C for two days before germination. The plates were positioned vertically, 45 °C from vertical, at 22 °C and 100 μmol/sec/m^2^ light intensity, in a climate control growth room with long day conditions (16h light, 8h dark).

### 4.2. Measurement of Root Length, Root Hair Traits and Gravitropic Index

After seven DAG the plates were scanned and total root length and gravitropic index were evaluated by using the ImageJ program from NIH. The gravitropic index was calculated according to [50]. Root tip bright field pictures for the evaluation of root hair traits were taken under the Zeiss880 microscope using the 20× objective and grabbing 3 × 9 tiles under 2× zoom, to capture 2 cm of the root tip. The root hair traits were also evaluated by using Image J. Distance to first root hair was calculated from the root tip to the first emerging root hair. Elongating root hairs were only taken into consideration when root hair length was measured. A summary of all average values with standard error and standard deviation is added in the Supplementary Tables S1 and S2). Statistical significance was assessed through unpaired Student’s *t*-tests for comparisons between two conditions (normally distributed data, GraphPad Prism QuickCalcs was used https://www.graphpad.com/quickcalcs/ttest1/) or Mann–Whitney *U* test (not normally distributed data, online tool—https://www.statskingdom.com/170median_mann_whitney.html); when more groups were evaluated against a control, Kruskal–Wallis test with Dunn’s post hoc test (online tool—https://astatsa.com/KruskalWallisTest/) was done. A summary of all average values with standard error and standard deviation is added in the Supplementary Figure S1.

## Data Availability

The data presented in this study are available in the Supplemental Table S4.

## Supplementary Materials

The following are available online at https://www.mdpi.com/2223-7747/10/1/111/s1, Figure S1A: example pictures of seedlings of all growth conditions, Figure S1B: example pictures of root hair outgrowth of all growth conditions, Table S1: descriptive statistics of analyzed root parameters, Table S2: descriptive statistics of analyzed root hair parameters, Table S3: comparison of all values relative to DGR with sucrose. Table S4: Raw data presented in this study.
