# Supplementary material for: Dissecting Hierarchies between Light, Sugar and Auxin Action Underpinning Root and Root Hair Growth
Source: Plants (Basel). 2021 Jan 7;10(1):111. doi: 10.3390/plants10010111 (PMC7826589; doi:10.3390/plants10010111)
Supplement: Supplementary file 1 [file plants-10-00111-s001.zip › plants-1047604-supplementary-xml.pdf]

Supplementary Data

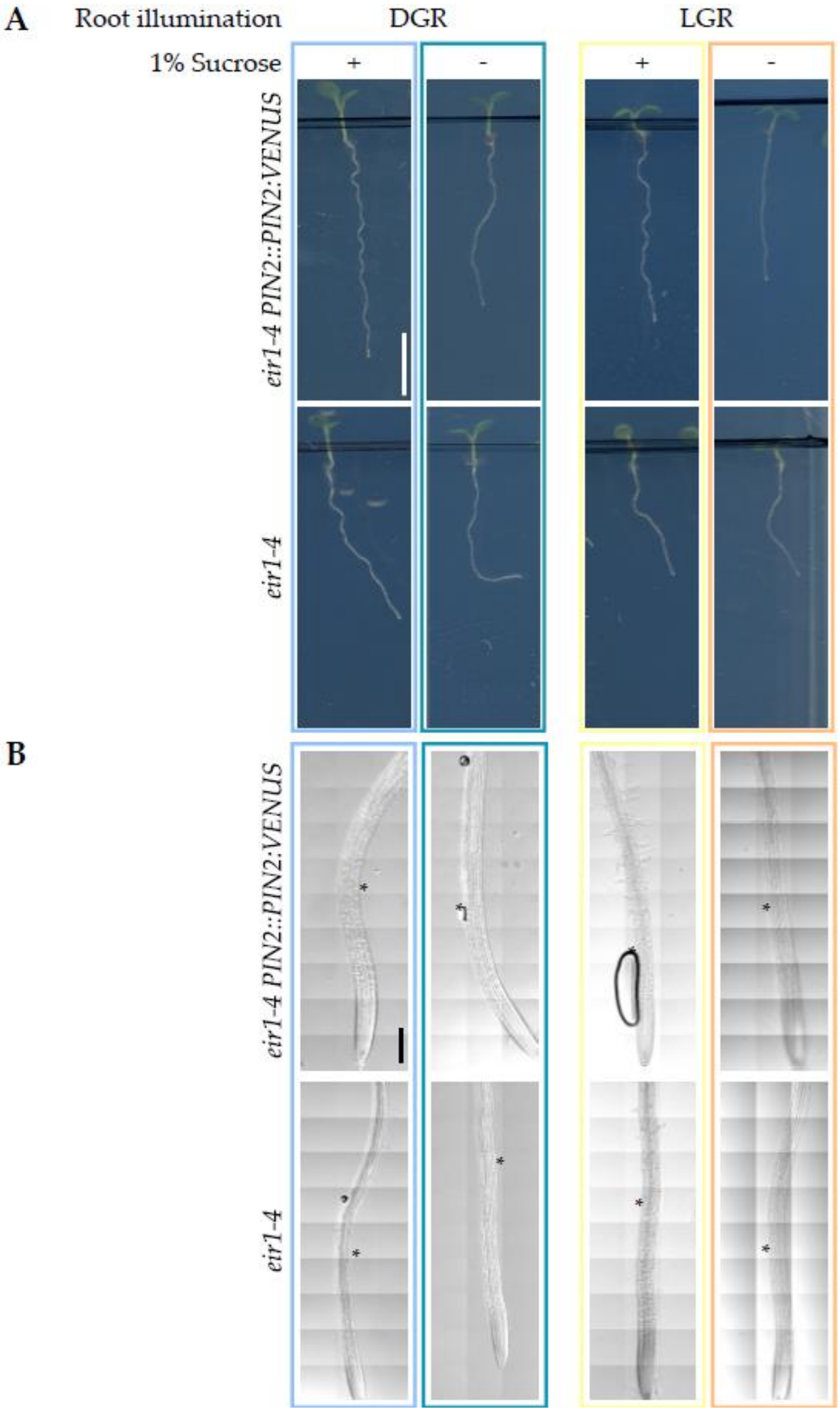

Figure S1 Representative images of seven-day-old seedlings of *Arabidopsis thaliana* plants grown in dark or light root illumination conditions in combination with sucrose supplementation. (A) Images of whole seedlings used for root parameter analysis (scale bar = 5mm). (B) Root tip images used for root hair evaluation. Roughly 2mm from the root tip were captured (scale bar = 200μm).

Table S1. Descriptive statistics of analyzed root parameters.

Mean ± SE/SD	LGR Sucrose	LGR No Sucrose	DGR Sucrose	DGR No Sucrose
Root length (mm)				
eir1-4 PIN2::PIN2:VENUS	16,71 ± 0,69/5,42	14,83 ± 0,74/5,94	19,53 ± 0,73/6,49	15,72 ± 0,72/5,74
eir1-4	14,67 ± 0,93/5,67	13,71 ± 1,03/6,18	17,33 ± 0,93/5,52	15,31 ± 0,94/5,48
GI (a.u.)				
eir1-4 PIN2::PIN2:VENUS	0,95 ± 0,003/0,03	0,97 ± 0,002/0,01	0,94 ± 0,003/0,023	0,97 ± 0,001/0,01
eir1-4	0,79 ± 0,027/0,17	0,82 ± 0,023/0,14	0,68 ± 0,038/0,23	0,83 ± 0,018/0,11

Table S2. Descriptive statistics of analysed root hair parameters.

Mean ± SE/SD	LGR Sucrose	LGR No Sucrose	DGR Sucrose	DGR No Sucrose
Total amount of root hairs				
eir1-4 PIN2::PIN2:VENUS	14,50 ± 2,44/8,46	9,25 ± 0,98/2,77	6 ± 0,93/2,45	5,75 ± 1,07/3,01
eir1-4	5,14 ± 0,79/2,96	2,75 ± 1,03/2,92	7,38 ± 1,21/3,42	1,75 ± 0,70/1,98
% elongating root hairs				
eir1-4 PIN2::PIN2:VENUS	75,20 ± 5,99/20,73	59,54 ± 11,85/24	63,8 ± 7,53/15,85	53,75 ± 9,66/27,34
eir1-4	60,98 ± 9,06/33,90	25,06 ± 12,53/35,43	70,88 ± 4,89/13,85	22,71 ± 11,61/32,83
Root hair length (μm)				
eir1-4 PIN2::PIN2:VENUS	66,29 ± 4,16/49,98	28,37 ± 3,26/21,88	20,47 ± 3,31/16,54	23,16 ± 3,22/16,43
eir1-4	35,28 ± 4,37/29,30	22,05 ± 6,11/22,84	44,04 ± 4,07/24,75	32,29 ± 8,30/21,97
Distance to first root hair (μm)				
eir1-4 PIN2::PIN2:VENUS	929,6 ± 91,87/304,7	950,4 ± 43,62/123,4	1043 ± 49,13/96,66	971,9 ± 56,25/159,25
eir1-4	1216 ± 74,08/277,2	1321 ± 126,3/357,3	1196 ± 101,3/286,5	1553 ± 138,4/391,5

Table S3. Root hairs of LGR grown on sucrose supplemented medium elongate more than three times longer in average compared to DGR grown on sucrose supplemented medium.

Root illumination	DGR				LGR			
1% Sucrose	+		-		+		-	
PIN2	*eir1-4 PIN2::PIN2:VENUS*	*eir1-4*	*eir1-4 PIN2::PIN2:VENUS*	*eir1-4*	*eir1-4 PIN2::PIN2:VENUS*	*eir1-4*	*eir1-4 PIN2::PIN2:VENUS*	*eir1-4*
Root length	100 +/- 5.28	88.75 +/- 5.81	80.50 +/- 4.75	78.40 +/- 5.73	85.53 +/- 4.78	75.11 +/- 5.54	75.93 +/- 4.74	70.20 +/- 5.89
Gravitropic index	100 +/- 0.39	72.18 +/- 4.07	103.10 +/- 0.32	87.77 +/- 1.94	100.33 +/- 0.45	84.05 +/- 2.89	103.16 +/- 0.34	86.49 +/- 2.42
Total amount of root hairs	100 +/- 21.82	122.92 +/- 27.67	95.83 +/- 23.10	29.16 +/- 12.52	241.67 +/- 55.20	85.71 +/- 18.67	154.17 +/- 28.83	45.83 +/- 18.58
% elongating root hairs	100 +/- 14.35	111.11 +/- 13.64	84.25 +/- 17.40	35.60 +/- 18.55	117.87 +/- 15.20	95.59 +/- 17.20	93.33 +/- 16.33	39.28 +/- 20.03
Distance to first emerging root hair	100 +/- 4.95	114.68 +/- 10.51	93.16 +/- 6.30	148.83 +/- 14.25	89.11 +/- 9.34	116.60 +/- 8.19	91.09 +/- 5.26	126.61 +/- 12.89
Root hair length	100 +/- 22.85	215.13 +/- 40.04	113.11 +/- 24.12	157.71 +/- 47.89	325.02 +/- 56.97	172.31 +/- 35.07	138.58 +/- 27.48	97.92 +/- 33.76
